# Crystal structure of Bcl-2 from lymphocystis disease virus 2 in complex with the BH3 domain of zebrafish BaxA

**DOI:** 10.71150/jm.2512006

**Published:** 2026-04-23

**Authors:** Dahwan Lim, So Hyeon Park, Joon Sig Choi, Ho-Chul Shin, Seung Jun Kim, Bonsu Ku

**Affiliations:** 1 Orphan Disease Therapeutic Target Research Center, Korea Research Institute of Bioscience and Biotechnology, Daejeon 34141, Republic of Korea; 2 Infectious Diseases Therapeutic Research Center, Korea Research Institute of Chemical Technology, Daejeon 34114, Republic of Korea; 3 Department of Biochemistry, Chungnam National University, Daejeon 34134, Republic of Korea; 4 Critical Diseases Diagnostics Convergence Research Center, Korea Research Institute of Bioscience and Biotechnology, Daejeon 34141, Republic of Korea

**Keywords:** lymphocystis disease virus 2, LCDV2, Bcl-2, zebrafish, BaxA, BH3, crystal structure

## Abstract

Lymphocystis disease viruses (LCDVs), members of the *Lymphocystivirus* genus of the *Iridoviridae* family, infect various freshwater and marine fish species. They cause the chronic disease lymphocystis, which is non-fatal, but substantially reduces the commercial value of the infected fish. To date, four genotypes of LCDV (LCDV1–4) have been identified, all of which encode the viral homologue of B-cell lymphoma 2 (Bcl-2), a key inhibitor of apoptosis. In this study, we performed biochemical and structural analyses of LCDV2 Bcl-2. Binding assays revealed that LCDV2 Bcl-2 exhibits binding selectivity toward BH3 domain-containing zebrafish proteins. It interacted with zBaxA and zNoxa, but not with zBaxB, zBid, or zBeclin 1, distinguishing it from mammalian and herpesviral Bcl-2 proteins. Subsequent structural determination of LCDV2 Bcl-2 in complex with the BH3 domain of zBaxA demonstrated that they interact in a canonical manner, primarily mediated by the BH3 consensus motif residues of zBaxA. In addition, a subpocket formed by two phenylalanine residues in LCDV2 Bcl-2 plays a key role in determining binding selectivity.

## Introduction

Lymphocystis disease viruses (LCDVs), members of the *Lymphocystivirus* genus of the *Iridoviridae* family, are double-stranded DNA viruses that infect diverse freshwater and marine fish species and cause chronic lymphocystis (Leiva-Rebollo et al., 2024; Tidona and Darai, 1997a). Lymphocystis is a benign disease that is typically non-fatal. However, the development of macroscopic nodules, primarily on the body surface and occasionally on internal organs, markedly reduces the commercial value of the infected fish (Leiva-Rebollo et al., 2024; Tidona and Darai, 1997a). To date, the complete genome sequences of LCDV1–4, the four LCDV genotypes classified by the International Committee on Taxonomy of Viruses, have been determined (Benkaroun et al., 2022; Doszpoly et al., 2020; Kawato et al., 2021; López-Bueno et al., 2016; Tidona and Darai, 1997b; Zhang et al., 2004). The genomes of LCDV1–4 range from 103 to 211 kb and contain 178–195 open reading frames (Benkaroun et al., 2022). Notably, 26 core genes are conserved across all four genotypes, encoding proteins involved in DNA replication, transcription, and virus assembly, as well as one apoptosis-associated protein, a viral homologue of B-cell lymphoma 2 (Bcl-2).

Bcl-2 family proteins are core regulators of apoptosis, a type I programmed cell death (Croce et al., 2025; Singh et al., 2019). Anti-apoptotic Bcl-2 family members, such as Bcl-2, Bcl-xL, and Mcl-1, contain a hydrophobic pocket, the BH3-binding groove (Kvansakul and Hinds, 2015). This pocket accommodates the Bcl-2 homology 3 (BH3) domain of pro-apoptotic members, such as Bak, Bim, and Noxa, thereby inhibiting apoptosis progression (Kvansakul and Hinds, 2015). Anti-apoptotic Bcl-2 family members also interact with and mediate the activity of several non-canonical BH3 domain-containing proteins, such as Beclin 1, a key regulator of autophagy that degrades and recycles damaged or unnecessary cellular components (Ku, 2023; Levine et al., 2008). Interestingly, Bcl-2 homologues have been identified in several viruses belonging to the families *Orthoherpesviridae*, *Asfarviridae*, *Poxviridae*, and *Iridoviridae* (Kvansakul and Hinds, 2013). Structural and biochemical investigations have revealed their three-dimensional structures and binding profiles with host BH3 domain-containing proteins, providing insights into how these viruses manipulate host cell apoptosis to evade degradation and promote viral replication (Kvansakul and Hinds, 2013; Suraweera et al., 2024). Although the functional aspects of viral Bcl-2 homologues in LCDVs have not yet been investigated, it is speculated that they act as anti-apoptotic and pro-proliferative factors in LCDV-associated lymphocystis in infected fish, based on two observations. First, the host anti-apoptotic Bcl-2 family protein Bcl-xL is significantly upregulated during lymphocystis formation (Zhang et al., 2023). Second, GIV66, a viral Bcl-2 protein of grouper iridovirus, which also belongs to the *Iridoviridae* family, inhibits apoptosis at an early stage of viral infection (Lin et al., 2008).

In this study, we conducted biochemical and structural analyses of the Bcl-2 homologue of LCDV2, referred to as LCDV2 Bcl-2, which has not yet been characterized. We identified the binding selectivity of the viral Bcl-2 protein and determined its crystal structure in complex with the BH3 domain derived from zebrafish BaxA. Structural analysis also revealed a subpocket composed of two phenylalanine residues in LCDV2 Bcl-2, which plays a key role in determining its binding selectivity.

## Materials and Methods

### Preparation, crystallization, and structural determination

The DNA fragment encoding LCDV2 Bcl-2 (residues 1–146) was cloned into a modified pET21 plasmid designed to express the protein with an N-terminal His_10_ tag (Novagen). The *Escherichia coli* BL21(DE3) RIL strain (Novagen) was cultured in Luria–Bertani medium at 25°C for 16 h for protein production. Protein expression was induced by 0.5 mM isopropyl β-D-thiogalactopyranoside treatment. Protein purification was performed using a Ni-NTA affinity chromatography column (QIAGEN) and a HiLoad 26/600 Superdex 75 prep grade size-exclusion chromatography column (Cytiva). The TEV protease was used to digest the His_10_ tag after Ni-NTA affinity chromatography. The final sample was equilibrated in a buffer consisting of 20 mM Tris-HCl (pH 8.0) and 150 mM NaCl. For complex formation, the LCDV2 Bcl-2 sample was mixed with a zebrafish BaxA(51–78) peptide at a 1:5 molar ratio and incubated at 4°C for 16 h. Crystals were obtained using the sitting-drop vapor diffusion method at 18°C by mixing and equilibrating 1 μl protein solution (10 mg/ml) and 1 μl precipitant solution containing 0.2 M ammonium citrate dibasic and 20% (w/v) polyethylene glycol 3350. The diffraction data collected using Beamline 11C at the Pohang Accelerator Laboratory (Korea) were processed with the *HKL*2000 program (Otwinowski and Minor, 1997). The complex structure was determined by molecular replacement using the Phaser program (McCoy et al., 2007) with the crystal structure of GIV66 (Protein Data Bank code: 5VMO) as the search model (Banjara et al., 2018). Model building and refinement were conducted using the Coot and PHENIX programs, respectively (Adams et al., 2010; Emsley and Cowtan, 2004). The crystallographic data are presented in Table 1.

### Isothermal titration calorimetry (ITC) measurements

Synthetic peptides of zebrafish BaxA (residues 51–78; wild-type, L61A∙L65A, D70A∙D73A, or A76Q), BaxB (residues 48–75), Bid (residues 82–109), Noxa (residues 3–29), and Beclin 1 (residues 99–126) were purchased from Dandicure (Korea). Peptides (0.5 mM) were titrated in 20 injections of 2 μl each against 200 μl of a His_10_−LCDV2 Bcl-2 (0.05 mM) solution containing 20 mM Tris-HCl (pH 8.0) and 150 mM NaCl. ITC experiments were performed once using a MicroCal Auto-iTC200 (MicroCal, Malvern-Panalytical), as detailed in our earlier studies (Jung et al., 2025; Lim et al., 2021).

## Results and Discussion

### Verification of the interaction between LCDV2 Bcl-2 and zebrafish proteins

**Fig. 1.**
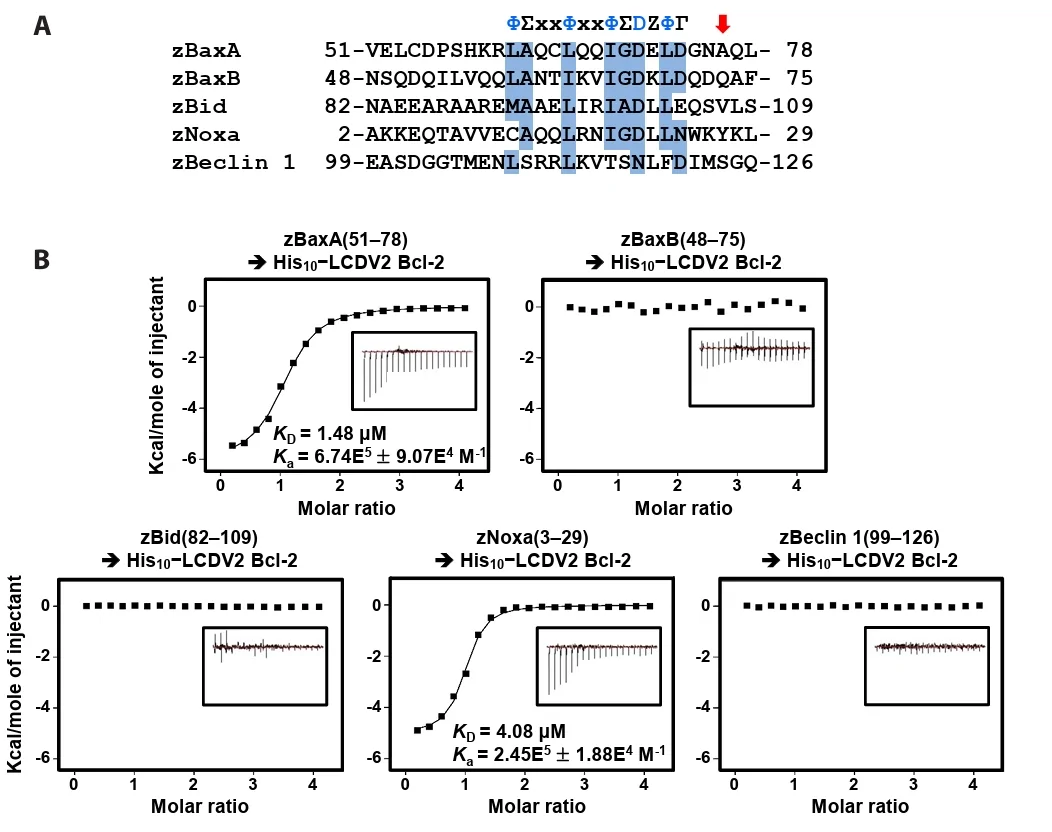
Interaction of LCDV2 Bcl-2 with zebrafish-derived BH3 peptides. (A) Sequence alignment of the five zebrafish-derived BH3 domains analyzed. Conserved residues are shaded in blue. The BH3 consensus motif is shown at the top: Ф, hydrophobic residue; ∑, small residue; Z, acidic residue; Γ, hydrophilic residue. The residue proposed to control the binding specificity of zBaxA and zBaxB for LCDV2 Bcl-2 (see Fig. 3C) is indicated by a red arrow. (B) ITC analysis. The indicated peptide (1 mM) was titrated into 50 μM recombinant His_10_-tagged LCDV2 Bcl-2, and the binding was analyzed using a VP-ITC MicroCalorimeter. *K*_D_ and *K*_a_ values were obtained from curve fitting of the integrated heat per mole of injected ligand.

To characterize the molecular interactions between LCDV2 Bcl-2 and host proteins, recombinant LCDV2 Bcl-2 was produced in the *E. coli* expression system. We also procured and prepared five synthetic peptides derived from the BH3 domain-containing proteins of zebrafish, a representative model fish species. The five zebrafish proteins, which are homologues of mammalian apoptosis- or autophagy-related proteins, are referred to as zBaxA, zBaxB, zBid, zNoxa, and zBeclin 1 in the present study (Kratz et al., 2006; Sasaki et al., 2014) (Fig. 1A). Next, we measured the binding affinities of the BH3 domain-containing peptides for LCDV2 Bcl-2 using ITC. LCDV2 Bcl-2 bound to zBaxA(51–78) and zNoxa(3–29) with dissociation constants (*K*_D_) of 1.48 μM and 4.08 μM, respectively, but showed no detectable interaction with zBaxB(48–75), zBid(82–109), or zBeclin 1(99–126) (Fig. 1B). These results indicated that LCDV2 Bcl-2 selectively recognizes the BH3 domains of zebrafish proteins.

Mammalian Bcl-2 proteins such as human Bcl-2 (Ku et al., 2011), and several viral Bcl-2 proteins from the *Orthoherpesviridae* and *Asfarviridae* families, including M11 of murine γ-herpesvirus 68 (Ku et al., 2008), KsBcl-2 of Kaposi’s sarcoma-associated herpesvirus (Suraweera et al., 2022), and A179L of African swine fever virus (ASFV) (Banjara et al., 2017), broadly interact with BH3 domains of diverse pro-apoptotic Bcl-2 family proteins and Beclin 1, a key autophagy regulator. Human Bcl-2 binds to the BH3 domains of 10 pro-apoptotic Bcl-2 family proteins and Beclin 1 with *K*_D_ values ranging from 2.4 to 1680 nM (Ku et al., 2008, 2011), and M11 of murine γ-herpesvirus 68 interacts with the BH3 domains of Bak, Bax, Bim, Bid, Puma, Bmf, Hrk, Noxa, and Beclin 1 with *K*_D_ values ranging from 40 to 719 nM, as determined by ITC (Ku et al., 2008). Thus, human and herpesvirus Bcl-2 proteins are considered “promiscuous” inhibitors of apoptosis and autophagy. In contrast, viral Bcl-2 proteins from the *Poxviridae* family (Kvansakul et al., 2008) and GIV66 of grouper iridovirus display binding specificity. For example, GIV66 binds to the BH3 domain of zebrafish Bim (zBim; *K*_D_ of 887 nM) but not to those of other pro-apoptotic Bcl-2 family proteins or zBeclin 1, as measured by ITC (Banjara et al., 2018). Similarly, our data showed that LCDV2 Bcl-2 exhibits selective binding toward certain BH3 domain-containing proteins (Fig. 1B). Therefore, we propose that LCDV2 Bcl-2 functions specifically in response to apoptotic stimuli involving zBaxA and zNoxa, but not in zBaxB- or zBid-associated apoptosis signaling or in the zBeclin 1-mediated autophagic pathway.

### Overall structure of LCDV2 Bcl-2 bound to zBaxA BH3

**Fig. 2.**
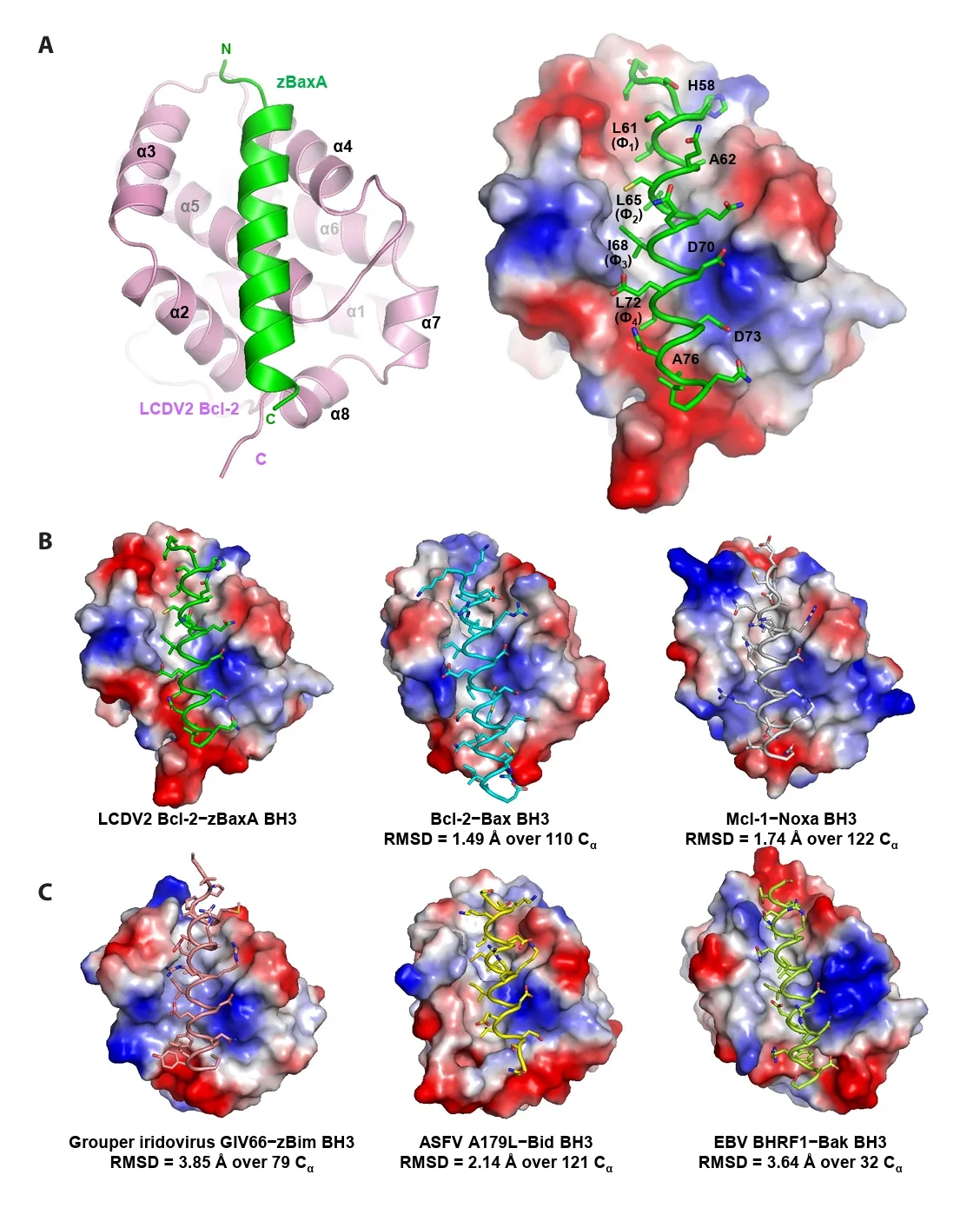
Crystal structure of LCDV2 Bcl-2 complexed with the zBaxA BH3 peptide. (A) Structural representation. (Left) LCDV2 Bcl-2 and the zBaxA BH3 peptide are shown as ribbon drawings with secondary structure elements labeled. (Right) LCDV2 Bcl-2 is displayed as an electrostatic surface representation in complex with the zBaxA BH3 peptide, whose side chains are shown as sticks. The zBaxA residues involved in binding to LCDV2 Bcl-2 are labeled. (B) Structural comparison of the LCDV2 Bcl-2−zBaxA BH3 complex with cellular or viral Bcl-2 homologues bound to the indicated BH3 peptides. The Bcl-2 homologues are shown as electrostatic surface representations, and the BH3 peptides as loops with side chains represented as sticks. The Protein Data Bank codes are 2XA0 for Bcl-2−Bax BH3, 2NLA for Mcl-1−Noxa BH3, 5VMO for GIV66−zBim BH3, 5UA5 for A179L−Bid BH3, and 2XPX for BHRF1−Bak BH3.

To gain atomic-level insights into the molecular association mediated by LCDV2 Bcl-2, we attempted to crystallize LCDV2 Bcl-2 incubated with BH3 domain-containing zBaxA(51–78) or zNoxa(3–29) peptides, guided by binding affinity measurements. Crystals of the two complexes were obtained; however, only the LCDV2 Bcl-2−zBaxA(51–78) complex crystals diffracted to high resolution, enabling structure determination at 2.3 Å by X-ray crystallography (Table 1). To the best of our knowledge, this is the first three-dimensional crystal structure of the Bcl-2 homologue from the *Lymphocystivirus* genus. Notably, this is the second Bcl-2 homologue structure from the *Iridoviridae* family, following GIV66 of grouper iridovirus, which belongs to the *Ranavirus* genus (Banjara et al., 2018). In the crystal structure, residues 58–77 of zBaxA adopt a single α-helix and are accommodated in the BH3-binding groove of monomeric LCDV2 Bcl-2, which is composed of eight α-helices (Fig. 2A). This represents a canonical association commonly observed between cellular or viral Bcl-2 proteins and BH3 domains. Structural superimposition showed that zBaxA(51–78)-bound LCDV2 Bcl-2 is homologous to mammalian antiapoptotic Bcl-2 family proteins, such as Bax BH3-bound Bcl-2 with a root mean square deviation (RMSD) value of 1.49 Å over 110 C_α_ atoms (Ku et al., 2011), and Noxa-bound Mcl-1 with an RMSD of 1.74 Å over 122 C_α_ atoms (Czabotar et al., 2007) (Fig. 2B, top). zBaxA(51–78)-bound LCDV2 Bcl-2 showed relatively lower structural similarity with BH3 fragment-bound viral Bcl-2 proteins, such as zBim BH3-bound grouper iridovirus GIV66 with an RMSD of 3.85 Å over 79 C_α_ atoms (Banjara et al., 2018), Bid BH3-bound ASFV A179L with an RMSD of 2.14 Å over 121 C_α_ atoms (Banjara et al., 2017), and Bak BH3-bound Epstein–Barr virus (EBV) BHRF1 with an RMSD of 3.64 Å over 32 C_α_ atoms (Kvansakul et al., 2010) (Fig. 2B, bottom).

### Analysis of the intermolecular interaction between LCDV2 Bcl-2 and zBaxA BH3

**Fig. 3.**
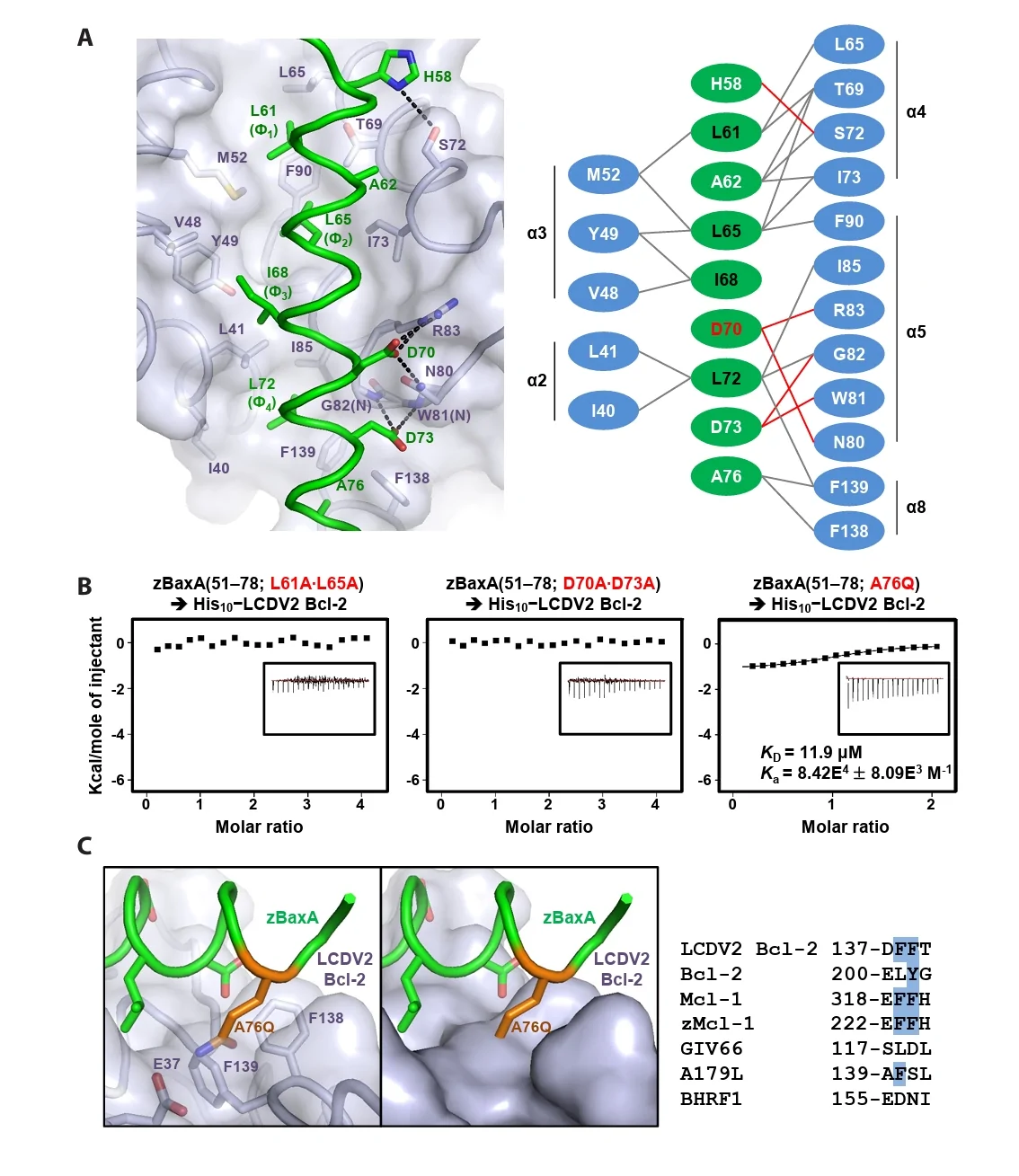
Molecular analysis of the interaction between LCDV2 Bcl-2 and zBaxA. (A) Intermolecular interactions. (Left) LCDV2 Bcl-2 and zBaxA are shown in light blue and green, respectively. Interacting residues are depicted as sticks with labels, and electrostatic interactions and hydrogen bonds are indicated by dashed lines. (Right) Binding network between LCDV2 Bcl-2 (blue) and zBaxA (green). Hydrophobic contacts are shown as gray lines, and electrostatic interactions and hydrogen bonds are indicated as red lines. The five BH3 consensus residues of zBaxA are highlighted in black (Leu61, Leu65, Ile68, and Leu72) or red (Asp70). Secondary structure elements of LCDV2 Bcl-2 are also indicated. (B) ITC analysis of zBaxA peptides containing structure-guided mutations highlighted in red. ITC experiments were performed using a VP-ITC MicroCalorimeter system with 50 μM His_10_-tagged LCDV2 Bcl-2 and 1 mM zBaxA(51–78; L61A∙L65A or D70A∙D73A) peptides, or a MicroCal Auto-iTC200 system with 100 μM His_10_-tagged LCDV2 Bcl-2 and 1 mM zBaxA(51–78; A76Q) peptide. (C) Structural modeling of the A76Q mutation in zBaxA and its effects on LCDV2 Bcl-2 binding. (Left) The A76Q substitution in zBaxA was predicted to cause steric hindrance with Phe138 and Phe139 of LCDV2 Bcl-2. (Right) Sequence alignment showing Phe138 and Phe139 of LCDV2 Bcl-2 compared with human, zebrafish, or viral Bcl-2 homologues. Conserved residues are shaded in blue. zMcl-1 denotes the zebrafish Mcl-1 homologue.

Next, we analyzed in detail the intermolecular interactions between LCDV2 Bcl-2 and the BH3 domain of zBaxA. The five BH3 consensus motif residues, including four hydrophobic residues and one aspartate residue, are well conserved in zBaxA BH3 as Leu61, Leu65, Ile68, Asp70, and Leu72 (Fig. 1A). In the LCDV2 Bcl-2−zBaxA BH3 complex structure, the four hydrophobic residues of zBaxA BH3 mediate tight hydrophobic interactions with multiple LCDV2 Bcl-2 residues, including Ile40 and Leu41 from α2; Val48, Tyr49, and Met52 from α3; Leu65, Thr69, and Ile73 from α4; Gly82, Ile85, and Phe90 from α5; and Phe139 from α8 (Fig. 3A). Moreover, additional hydrophobic contacts are formed between Ala62 and Ala76 of zBaxA and several LCDV2 Bcl-2 residues, including Thr69, Ser72, and Ile73 from α4 and Phe138 and Phe139 from α8 (Fig. 3A). In addition, intermolecular electrostatic interactions and hydrogen bonds further stabilize this complex. An electrostatic interaction was observed between the negatively charged carboxyl group of Asp70 in zBaxA, which is one of the five BH3 consensus motif residues, and the positively charged guanidinium group of Arg83 in LCDV2 Bcl-2 (Fig. 3A). Hydrogen bonds are established between the side chains of His58, Asp70, and Asp73 of zBaxA and those of Ser72, Asn80, and Arg83, as well as the main chain amides of Trp81 and Gly82 of LCDV2 Bcl-2 (Fig. 3A). To examine the physiological significance of these interactions in complex formation, we prepared two zBaxA(51–78) mutant peptides: L61A∙L65A and D70A∙D73A, which are expected to disrupt hydrophobic interactions and electrostatic interactions/hydrogen bonds, respectively. Neither of the two peptides bound to LCDV2 Bcl-2 in ITC measurements (Fig. 3B, left and middle), demonstrating that both nonpolar and polar associations are required for binding between LCDV2 Bcl-2 and the zBaxA BH3 domain.

In the ITC measurements shown in Fig. 1, LCDV2 Bcl-2 interacted with zBaxA(51–78) but not with zBaxB(48–75), even though the five BH3 consensus residues are well conserved in both proteins. Based on the LCDV2 Bcl-2−zBaxA(51–78) complex structure, we hypothesized that Phe138 and Phe139, which are not conserved in Bcl-2 homologues except for Mcl-1 (Fig. 3C, right), are critical determinants of the binding specificity of LCDV2 Bcl-2 for zBaxA over zBaxB. The aromatic rings of these two phenylalanine residues form a subpocket that interacts with the side-chain methyl group of Ala76 in zBaxA (Fig. 3A). In contrast, zBaxB harbors Gln73 at the corresponding position (Fig. 1A), and Phe138 and Phe139 of LCDV2 Bcl-2 were predicted to cause severe steric clashes with Gln73 of zBaxB, as observed in the LCDV2 Bcl-2−zBaxA(51–78; A76Q) complex model (Fig. 3C, left). To test this hypothesis, we performed an additional ITC analysis. Introduction of the A76Q substitution into the zBaxA(51–78) peptide caused an approximately eight-fold reduction in its binding affinity for LCDV2 Bcl-2, from 1.48 μM (Fig. 1B) to 11.9 μM (Fig. 3B, right), highlighting the importance of this subpocket of LCDV2 Bcl-2 for zBaxA binding. Taken together, these results imply that Gln73 of zBaxB prevents this protein from interacting with LCDV2 Bcl-2 and that LCDV2 Bcl-2 employs two phenylalanine residues to confer selectivity towards its binding partners.

Although four highly conserved hydrophobic residues in the BH3 consensus motif serve as key determinants mediating intermolecular interactions between Bcl-2 family proteins and BH3 domains, several structural studies have revealed additional nonpolar residues in some BH3 domains that contribute to complex formation. Tandem isoleucine residues preceding consensus residues play a substantial role in binding between Bak and the BH3 domain of Pxt1 or Bnip5 (Aguilar et al., 2023; Lim et al., 2023, 2024). Puma, another pro-apoptotic Bcl-2 family member containing a BH3 domain, harbors a hydrophobic residue (Tyr152) located downstream of the BH3 consensus residues, which provides additional hydrophobic interactions that stabilize complex formation with Mcl-1 and KsBcl-2 (Suraweera et al., 2022). Our structural and biochemical data demonstrated that noncanonical hydrophobic contacts between Ala76 of zBaxA and Phe138 and Phe139 of LCDV2 Bcl-2 might contribute to complex formation and binding selectivity. Given that the two phenylalanine residues are conserved in Mcl-1 (Fig. 3C), this protein may likewise distinguish between zBaxA and zBaxB in binding, which requires further investigation.

### Analysis of BH3-binding interactions of LCDV Bcl-2 homologues

**Fig. 4.**
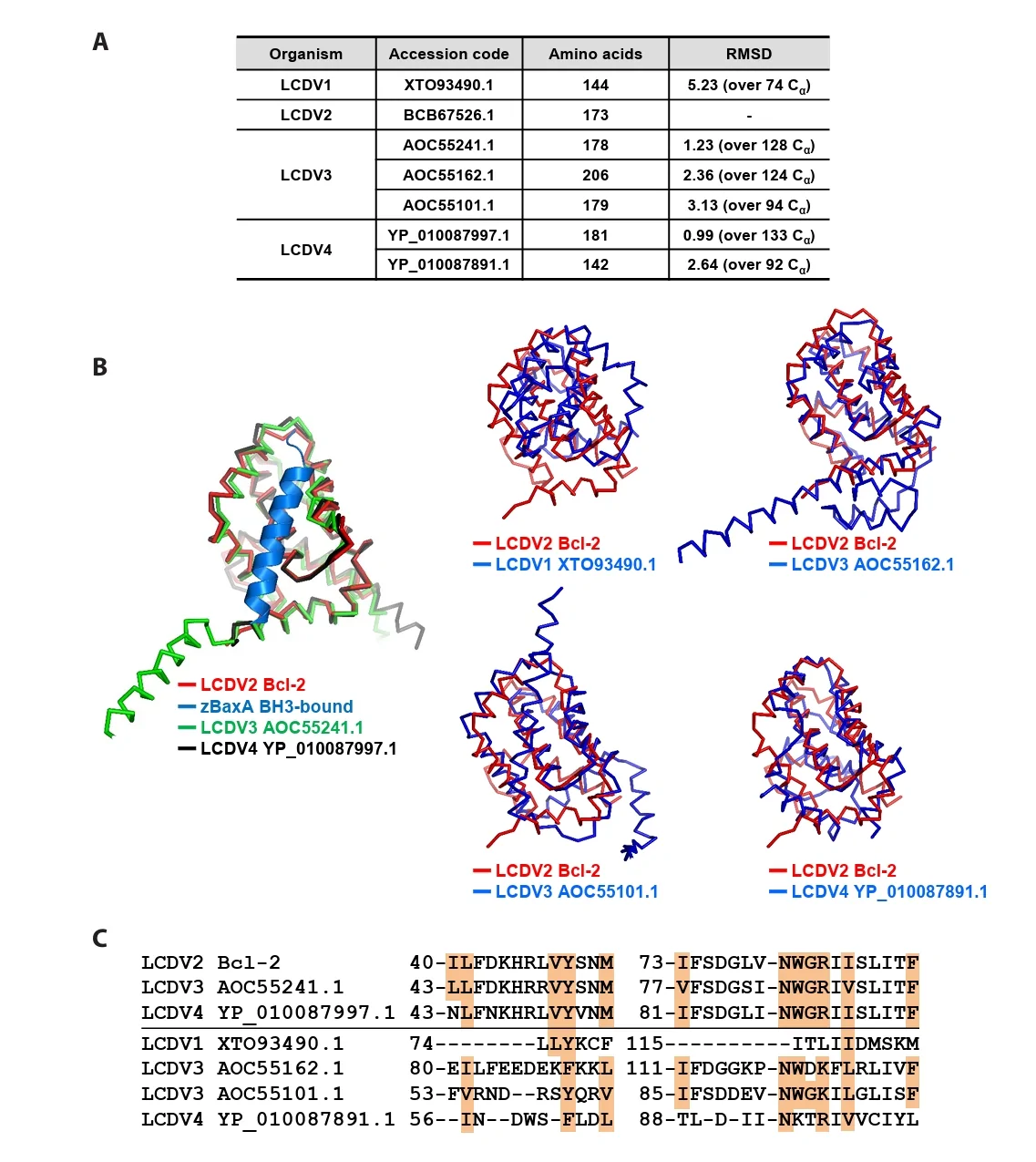
Structural and sequence alignment of LCDV Bcl-2 homologues. (A) List of LCDV Bcl-2 homologues. Accession codes correspond to entries in the NCBI database. RMSD values were calculated relative to LCDV2 Bcl-2. (B) Structural alignment of LCDV Bcl-2 homologues. Three-dimensional structures were modeled using AlphaFold 3, except for LCDV2 Bcl-2 bound to the zBaxA BH3 domain determined by X-ray crystallography. (C) Sequence alignment. Conservation of the zBaxA BH3 binding residues of LCDV2 Bcl-2 among LCDV Bcl-2 homologues is indicated by shading.

To date, seven Bcl-2 homologues have been identified in the genomes of LCDV1–4 (Fig. 4A). When their three-dimensional structures were modeled using AlphaFold 3 and then superimposed onto the crystal structure of LCDV2 Bcl-2, we found that two viral proteins (AOC55241.1 of LCDV3 and YP_010087997.1 of LCDV4) share high structural similarity with LCDV2 Bcl-2 (RMSDs of 1.23 Å and 0.99 Å, respectively) and are predicted to bind the BH3 domain of zBaxA well, whereas the remaining homologues exhibit relatively low structural homology (RMSDs of 2.36–5.23 Å) (Fig. 4B). Moreover, the LCDV2 Bcl-2 residues involved in zBaxA BH3 binding (Fig. 3) are mostly conserved in AOC55241.1 of LCDV3 and YP_010087997.1 of LCDV4, but only partially conserved in the other homologues (Fig. 4C). Collectively, we propose that targeting zBaxA and zNoxa via viral Bcl-2 homologue proteins represents a common strategy in at least LCDV2–4. The precise functional roles and binding partners of Bcl-2 homologues structurally distinct from LCDV2 Bcl-2, found in LCDV1, LCDV3, and LCDV4, warrant further investigation.

## Conclusion

In this study, we characterized the molecular features of LCDV2 Bcl-2 by identifying its binding partners, zBaxA and zNoxa, and determining and analyzing its crystal structure in complex with the zBaxA BH3 domain. Our findings provide the molecular basis for understanding how LCDV2 targets host proteins and modulates apoptosis to promote cell survival and viral replication. We believe that this information will be valuable for developing therapeutic strategies against this virus, which is the causative agent of chronic lymphocystis in freshwater and marine fish.

## Figures and Tables

**Table 1.** Data collection and structure refinement statistics

Protein	LCDV2 Bcl-2−zBaxA(51–78)
**Data collection**	
Space group	*P*4_1_2_1_2
Unit cell dimensions	
a, b, c (Å) / a = β = γ (°)	89.14, 89.14, 50.60 / 90
Resolution (Å)	50.0−2.3 (2.34−2.30)^a^
*R*_sym_^b^ (%)	11.6 (30.1)
*I / σ* (*I*)	26.3 (3.9)
Completeness (%)	96.8 (94.6)
Redundancy	5.5
**Refinement**	
Resolution (Å)	50.0−2.3
Number of reflections	9304
*R*_work_^c^ / *R*_free_ (%)	19.3 / 24.3
Number of atoms	
Protein	1133
Peptide	176
Water	28
RMSD	
Bond lengths (Å)	0.006
Bond angles (°)	0.728
Ramachandran plot (%)	
Most favored region	96.9
Additionally allowed region	3.1
Average B-values (Å^2^)	
Protein	43.5
Peptide	46.5
Water	45.6

a The numbers in parentheses are statistics from the shell with the highest resolution.

b *R*_sym_ = Σ |*I*_obs_ - *I*_avg_| / *I*_obs_, where *I*_obs_ is the observed intensity of individual reflection and *I*_avg_ is the average across symmetry equivalents.

c *R*_work_ = Σ ||*F*_o_| - |*F*_c_|| / Σ |*F*_o_|, where |*F*_o_| and |*F*_c_| are the observed and calculated structure factor amplitudes, respectively. *R*_free_ was calculated with 10.0% of the data.

## Data Availability

The coordinates of the LCDV2 Bcl-2−zBaxA(51–78) structure, together with the structure factors, were deposited in the Protein Data Bank under accession code 21CP.
